# Comparison of intraocular pressure as measured by three different non-contact tonometers and goldmann applanation tonometer for non-glaucomatous subjects

**DOI:** 10.1186/s12886-017-0593-1

**Published:** 2017-11-02

**Authors:** Seung Pil Bang, Chong Eun Lee, Yu Cheol Kim

**Affiliations:** Department of Ophthalmology, Keimyung University School of Medicine, Dongsan Medical Center, #56, Dalseong-ro, Jung-gu, Daegu 41931 South Korea

**Keywords:** Central corneal thickness, Goldmann applanation tonometer, Intraocular pressure, Non-contact tonometer

## Abstract

**Background:**

To compare the measurement of intraocular pressure (IOP) among the three different non-contact tonometers (NCT) and the Goldmann applanation tonometer (GAT) for non-glaucomatous subjects.

**Methods:**

In 52 eyes of 52 non-glaucomatous subjects, IOP was measured sequentially with the Canon TX-20P, the Nidek NT-530P, the Topcon CT-1P, and the GAT at the same time. We evaluated the IOP-measurement agreement among the tonometers as well as the factors affecting the measurements.

**Results:**

A significant positive correlation was shown between the IOP values obtained with GAT and each NCT. The Canon TX-20P showed statistically the most significant agreement with the GAT (ICC 0.906, 95% CI 0.837–0.946). In an analysis of the Bland-Altman plots, the Canon TX-20P also showed the largest mean bias (1.38 mmHg) but the narrowest limits of agreement (LoA) (95% LoA; ± 3.43 mmHg). The Topcon CT-1P showed the smallest mean bias (0.48 mmHg) but the widest LoA (95% LoA; ± 4.16 mmHg). The Topcon CT-1P and Nidek NT-530P both showed a significantly positive correlation between increasing central corneal thickness (CCT) and increasing IOP.

**Conclusion:**

There was a statistically significant correlation between each of the three different NCT and the GAT measurements. IOP measured with the Canon TX-20P and Topcon CT-1P tended to be higher, and with the Nidek NT-530P lower, than with the GAT. Practitioners need to know the properties of their own NCTs and their respective measurement tendencies.

## Background

Currently, the Goldmann applanation tonometer (GAT) is considered to be the gold standard for IOP measurement in clinical practice [1–6]. However, since their introduction, non-contact tonometers (NCTs) have become well established in clinical practice. NCT is a rapid, simple and objective method of intraocular pressure (IOP) measurement that can be performed by ancillary staff without the use of corneal anaesthesia [7].

Not all NCTs perform similarly. Although earlier models showed, relative to the GAT, a wider bias and a poor sensitivity for detection of elevated IOP [1, 3, 7, 8], the accuracies of subsequent models are much improved [9–18]. The Canon TX-20P, Nidek NT-530P, and Topcon CT-1P are the new automatic tonometers currently marketed. Perhaps surprisingly, there are in the literature, to our knowledge, few reports available on the performance of the Canon TX-20P or Nidek NT-530P [10–15], and none on the Topcon CT-1P. Also, various studies have compared just one or two of the NCTs with the GAT, and there have been few studies comparing the accuracy of various tonometers for non-glaucomatous healthy adult subjects [10, 14, 19–21].

The purposes of this study were to evaluate the reliability of the three new NCTs and to compare their IOP measurements with those obtained by the GAT for non-glaucomatous subjects. For each tonometer, the relationships between the measured ocular parameters and the IOP measurements also were examined.

## Methods

Fifty-two eyes of 52 non-glaucomatous healthy subjects aged 21 to 85 years were included in the study. The study was approved by the Institutional Review Board (IRB) of Keimyung University Dongsan Medical Center (IRB no. 2015–12-052) and was performed in accordance with the tenets of the Declaration of Helsinki. None of the subjects had any history of ocular pathology affecting IOP, refractive surgery or trauma, and none had worn contact lenses within the two-week period prior to IOP measurement. Subjects were excluded if they had regular astigmatism greater than 3.50 diopters (D) or any irregular astigmatism. To avoid the double-organ bias, one eye per individual was randomly included in the analysis using a table of random numbers for randomization [22]. In a fixed sequence, all of the subjects were examined with the three NCTs, the GAT and ultrasound pachymeter (850, Humphrey Instruments, Inc., San Leandro, CA, USA) to obtain IOP and central corneal thickness (CCT) measurements, respectively. According to the GAT IOP values, the eyes were categorized into low-teen (<14 mmHg), mid-teen (≥14 and ≤17 mmHg) and high-teen (>17 mmHg) groups.

IOP measurements were made by the same experienced ancillary staff using the three NCTs. The order in which the instruments were used was the Canon TX-20P, followed by the Nidek NT-530P, the Topcon CT-1P and the GAT. Each of the tonometers was calibrated according to the manufacturer’s guidelines prior to its use in this study. In manual measurements using the Nidek NT-530P, on the other hand, the operator aligns the cornea by superimposing a reflection of the target from the subject’s cornea on a stationary ring and depresses the trigger when the cornea is aligned. In the present study, the mean of three measurements was used so as to avoid the effect of fluctuations caused by the cardiac pulse cycle.

IOP measurements were taken with the GAT (AT900; Haag-Streit, Köniz, Switzerland) according to the standard procedures. Before acquisition, one drop of 0.5% proparacaine hydrochloride eye drops (Alcaine®, Alcon Laboratories Inc., Fort Worth, TX, USA) was instilled and a fluorescein strip was applied to the inferior conjunctival fornix. To avoid error introduced by topical anesthesia, the GAT was applied five minutes after eyedrop instillation [23]. The last IOP measurement was obtained using GAT to avoid a corneal-compression-induced aqueous outflow increase that would have affected subsequent IOP readings [24, 25]. Also, the mean of three measurements of GAT was used and each IOP readings were masked to the one clinician (SPB) performing the measurements. Between each instrumentation application, the subjects were allowed a five-minute rest period to recover from the aqueous outflow. All of the measurements were taken between 11:30 am and 1:30 pm in order to minimize the effects of diurnal IOP variation [26].

Pearson correlation analysis, the intraclass correlation coefficient (ICC), the paired t-test were used to assess the correlation, consistency and agreement among the IOP measurements provided by each instrument. We also constructed Bland-Altman plots using Medcalc version 15.2 (Ostend, Belgium) to compare the bias in the IOP measurements of each NCT relative to the GAT. Simple linear regression analysis was used to assess the correlations between CCT and the IOP measurements of each tonometer. The paired t-test was used to analyze the difference between the IOP measurements of each NCT and the GAT in each subgroup divided by the GAT IOP values. Data were analyzed using SPSS version 22 (IBM, Armonk, NY, USA).

## Results

Of the 52 participants, 22 were male and 30 were female, with a mean age of 50.56 ± 17.25 years (range: 21–85 years). The mean IOP across all subjects was 17.23 ± 2.94 mmHg (range: 11–21 mmHg) with the Canon TX-20P, 14.87 ± 3.25 mmHg (range: 8–21 mmHg) with the Nidek NT-530P, 16.33 ± 3.01 mmHg (range: 9–21 mmHg) with the Topcon CT-1P, and 15.85 ± 3.05 mmHg (range: 8–21 mmHg) with the GAT. The mean CCT as measured with the ultrasound pachymeter was 541.44 ± 28.49 μm (range: 469–605 μm).

### IOP comparisons

**Fig. 1:**
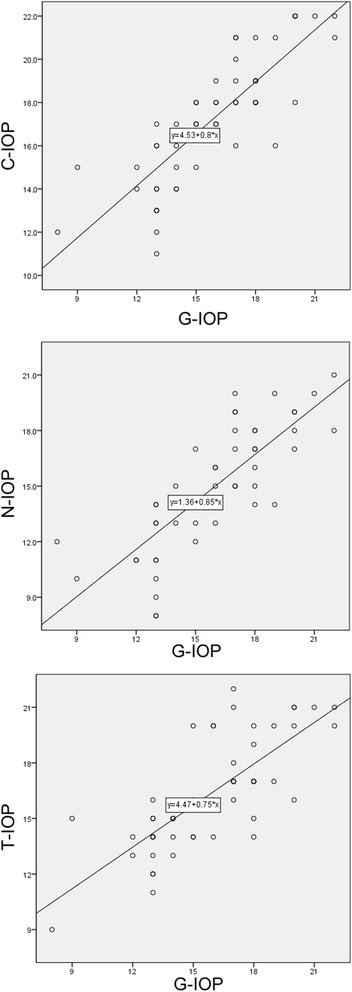
Correlation analysis of intraocular pressure (IOP) measurements (mmHg) taken with three different non-contact tonometers (NCTs) and Goldmann applanation tonometer (GAT). Significant positive correlations by Pearson correlation analysis were noted between the IO*P* values measured by each of the NCTs and the GAT. C-IOP = IOP value measured by Canon NCT; N-IOP = IOP value measured by Nidek NCT; T-IOP = IOP value measured by Topcon NCT; G-IOP = IOP value measured by GAT

A significant positive correlation was shown between the IOP values obtained with the GAT (G-IOP) and the Canon TX-20P (C-IOP: *R* = 0.829, *p* < 0.001), the Nidek NT-530P (N-IOP: *R* = 0.799, *p* < 0.001) and the Topcon CT-1P (T-IOP: *R* = 0.755, *p* < 0.001), respectively (Fig. 1). Also, a significantly high consistency by ICC was observed between the G-IOP and the C-IOP (ICC = 0.906, 95% CI: 0.837–0.946, *p* < 0.001), the N-IOP (ICC = 0.887, 95% CI: 0.803–0.935, p < 0.001) and the T-IOP (ICC = 0.861, 95% CI: 0.757–0.920, p < 0.001), respectively.

**Fig. 2:**
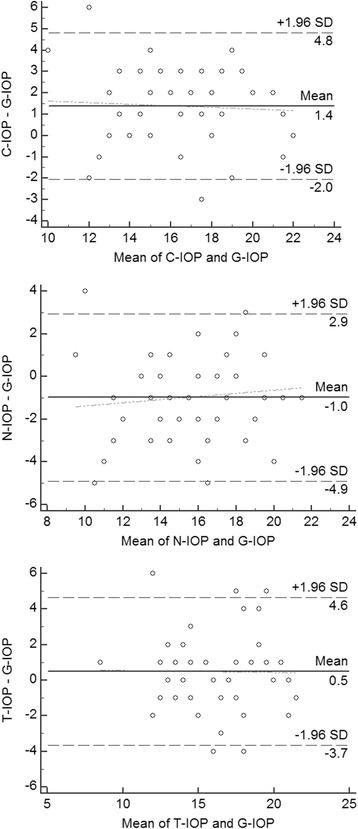
Bland-Altman plots between IOP measurements of each NCT and GAT, as plotted against mean of two measurements for each subject together with mean difference and 95% confidence limits. The solid line indicates the mean difference of both tonometers. The dashed lines are the 95% limits of agreement (LoA). The dotted line indicates equality between tonometers, showing good equality between the 3 NCTs and GAT. C-IOP: IO*P* value measured by Canon NCT; N-IOP: Nidek NCT; T-IOP: Topcon NCT; G-IOP: GAT

Figure 2 shows a Bland-Altman scatterplot comparing the three NCT and GAT readings. The mean of the differences between the C-IOP and G-IOP was 1.38 ± 1.75 mmHg (95% LoA, −2.05 to +4.82 mmHg), between the N-IOP and G-IOP, −0.98 ± 2.00 mmHg (95% LoA, −4.91 to +2.95 mmHg), and between the T-IOP and G-IOP, 0.48 ± 2.12 mmHg (95% LoA, −3.67 to +4.63 mmHg). The narrowest 95% LoA, as indicative of the highest consistency, was that between the C-IOP and G-IOP. There was no significant linear relationship between the difference and the mean of each NCT/GAT pair, which fact indicated good equality between the 3 NCTs and the GAT.

There was a significant mean difference between, respectively, the Canon TX-20P and Nidek NT-530P and GAT IOP measurements but not between the Topcon CT-1P and GAT measurements. The mean difference between the C-IOP and G-IOP was 1.38 mmHg (*p* < 0.001), and between the N-IOP and G-IOP, 0.98 mmHg (*p* = 0.001), as noted above (Table 1). There was no significant mean difference between the IOP measurements of each NCT and those of the GAT in six of the nine subgroups divided by G-IOP; significant differences were found in the low- and mid-teen groups of the C-IOP and in the high-teen group of N-IOP. The mean differences between the C-IOP and G-IOP were 1.71 mmHg (*p* = 0.013) in the low-teen group and 1.77 mmHg (*p* < 0.001) in the mid-teen group. The mean difference between the N-IOP and G-IOP was 1.75 mmHg (*p* = 0.001) in the high-teen group (Table 2).

**Table 1:** Pairwise comparative analyses between each NCT and GAT

NCT device	Mean (±SD) (mmHg)	Mean difference (±SD) with G-IOP	95% LoA	*p* value
C-IOP	17.23 ± 2.94	1.38 ± 1.75	−2.05 to 4.82	**<0.001**
N-IOP	14.87 ± 3.25	−0.98 ± 2.00	−4.91 to 2.95	**0.001**
T-IOP	16.33 ± 3.01	0.48 ± 2.12	−3.67 to 4.63	0.108

*C-IOP* IOP value measured by Canon NCT, *N-IOP* IOP value measured by Nidek NCT, *T-IOP* IOP value measured by Topcon NCT, *G-IOP* IOP value measured by GAT, *LoA* limits of agreement

P value by paired t-test

**Table 2:** Pairwise comparative analyses between each NCT and GAT in subgroups divided by G-IOP

Subgroups^a^	n	C-IOP – G-IOP	N-IOP – G-IOP	T-IOP – G-IOP
Low-teen	14	**1.71 ± 2.23 (** ***p*** **= 0.013)**	−1.14 ± 2.57 (*p* = 0.120)	1.14 ± 1.96 (*p* = 0.048)
Mid-teen	22	**1.77 ± 1.34 (** ***p*** **< 0.001)**	−0.32 ± 1.67 (*p* = 0.382)	1.00 ± 2.07 (*p* = 0.034)
High-teen	16	0.56 ± 1.59 (*p* = 0.178)	**−1.75 ± 1.65 (** ***p*** **= 0.001)**	−0.81 ± 1.83 (*p* = 0.097)

*C-IOP* IOP value measured by Canon NCT, *N-IOP* Nidek NCT, *T-IOP* Topcon NCT, *G-IOP* GAT

Values are presented as the mean ± standard deviation. P value by paired t-test

^a^Categorized into low-teen (<14 mmHg), mid-teen (≥14 and ≤17 mmHg) and high-teen (>17 mmHg) groups

### Influence of CCT

There was a significant positive correlation between CCT and T-IOP (*R* = 0.390, *p* = 0.004), the IOP increasing with increasing corneal thickness; linear regression analysis showed a mean change of 0.4 mmHg in T-IOP per 10 μm variation in CCT. Meanwhile, there was a weak positive correlation between CCT and N-IOP (*R* = 0.325, *p* = 0.019); linear regression analysis also showed a mean change of 0.4 mmHg in N-IOP per 10 μm variation in CCT. Contrastingly, no correlation was detected between CCT and C-IOP (*R* = 0.237, *p* = 0.091) or G-IOP (*R* = 0.149, *p* = 0.293), indicating no significant effect of CCT on C-IOP or G-IOP.

## Discussion

This study compared the IOP-measurement performances of the three new NCTs with the GAT. With the same subjects at the same moment, the IOPs from each NCT were obtained and demonstrated the following tendencies in terms of range: the IOP measurements with the Canon TX-20P and the Topcon CT-1P tended to be higher, whereas those with the Nidek NT-530P tended to be lower than with the GAT. Considering the order of the tonometer measurements, the greatest IOP values of C-IOP could be due to the ‘massage effect’, though the NCTs were less affected than was the GAT [24, 25], and regardless, the five-minute interval between each measurement, enough time to recover from enhanced aqueous outflow due to corneal compression, would minimize such effect.

In terms of study samples, the mean IOPs recorded using the four tonometers were close to those obtained by other authors for healthy subjects (15–16 mmHg on average, SD 2.5–3.0 mmHg) [3, 27]. Similarly, the mean CCT of 541.4 μm determined using the ultrasound pachymeter was close to the population mean of 530.9 μm and within the range found in normal Korean subjects [28].

The relationships observed between the CCT and IOP measurements obtained by the Nidek NT-530P and Topcon CT-1P tonometers are in agreement with the general assumption that CCT affects NCT readings, showing a positive correlation. According to previous reports, CCT affects both GAT and NCT readings [29, 30], the latter being more affected than the former [30–32]. The moderate correlations observed in this study (*R* = 0.325 and 0.390) could be attributed to the limited range of CCT in our population (469–605 μm). Other authors argue that for corneas with a CCT less than 575 μm, IOP measurements are unaffected [5, 33]. In our study population, only 6 of the 52 eyes showed a CCT greater than 575 μm.

Through Pearson correlation analysis (Fig. 1), we observed a highly significant relationship between the pressure readings provided by each NCT and those offered by the GAT, confirming the good relationships between NCT- and GAT-determined IOPs reported by others [33]. These results indicate that the three NCTs provide statistically significant predictions of G-IOP measurements.

Many variables affect IOP measurements, including astigmatism, corneal thickness, corneal biomechanics/hysteresis, accommodation, respiration, heart rate and rhythm [34], and diurnal variation [26]. Considering these sources of error, some authors advocate that ±3.0 mmHg should be the maximum clinically acceptable error [19]. In our study, according to the Bland-Altman plots, the 95% LoA between C-IOP and G-IOP were close to this clinically acceptable error (±3.43 mmHg), 92% of the differences falling within this range. This means that the N-IOP showed 8% clinical error in our study subjects, all of whose IOP was within the normal range. In case of N-IOP, the LoA were wider (±3.92 mmHg), and 89% of the differences were within the clinically acceptable limits. The LoA of T-IOP was the widest (±4.16 mmHg), and 87% of the differences were within the clinically acceptable limits. These differences of LoA among the three NCTs might be accounted for in the differences in the alignment methods: the Canon TX-20P and Topcon CT-1P make parallel automatically, whereas the Nidek NT-530P aligns manually. Manual alignments might allow the operator to more accurately adjust subjects’ cornea in the three, axial, vertical and lateral dimensions. The reason for the narrower LoA of the Canon TX-20P than that of the Nidek NT-530P should be clarified by further evaluation with a larger subject cohort.

While there was a statistically significant difference between C-IOP and G-IOP as well as between N-IOP and G-IOP, none such was detected between T-IOP and G-IOP, suggesting that any such differences as might exist are independent of the mean IOP values. Thus, we could argue that the Topcon CT-1P offers relatively closer agreement with the GAT than does the Canon TX-20P or Nidek NT-530P. Furthermore, in the subgroup comparison study by G-IOP, the low- and mid-teen subgroups between C-IOP and G-IOP and the high-teen subgroup between N-IOP and G-IOP showed significant mean differences, demonstrating the tendency of discrepancy between the Canon TX-20P and GAT as well as between the Nidek NT-530P and GAT in these G-IOP ranges.

There are some limitations to this study. The fact that we did not test the instruments across the entire useful range is the main one. We also excluded subjects with irregular astigmatism or astigmatism of more than 3.5 diopters, and thus, our findings may hold true only for subjects with similar refraction characteristics. Furthermore, the sample size of this study was relatively small for the subgroup analysis; future studies will need to include larger populations of ocular hypertensive and glaucoma subjects, with or without irregular/severe astigmatism, for whom any bias between instruments could have clinical implications. Additionally, we were unable to randomize the order in which the NCTs were used due to restraints on access to the equipment. Therefore, we were unable to avoid potential systematic errors and are unable to ensure that the lack of randomization did not influence the outcome. We did implement a five-minute interval between each measurement to minimize the ‘massage effect’. One other limitation was the fact that we did not carry out CCT correction separately with each tonopachymeter; future studies should also investigate the results of CCT correction and comparison of CCT values from the ultrasound pachymeter with those from each tonopachymeter.

In conclusion, this comparative study shows that the only Canon TX-20P provides reliable and repeatable IOP measurements not influenced by CCT within a relatively normal/restricted range of corneal thickness. However, without CCT correction, the Canon TX-20P, Nidek NT-530P and Topcon CT-1P all offer similar accuracy to the GAT. Despite the relatively small sample size of this study and the absence of high IOPs, the instruments demonstrated good performance in measuring IOP in ocular normotensive non-glaucomatous subjects. These results suggest the instruments will prove useful for rapid IOP testing in clinical practice. Indeed, practitioners should keep in mind the differences in the tendencies of the IOP values as measured by the respective NCTs.

## Conclusion

Without CCT correction, the Canon TX-20P, Nidek NT-530P and Topcon CT-1P all offer similar accuracy to the GAT.

## Abbreviations

C-IOP: Intraocular pressure values obtained with the Canon TX-20P
CCT: Central corneal thickness
D: Diopters
GAT: Goldmann applanation tonometer
G-IOP: Intraocular pressure values obtained with the GAT
ICC: Intraclass correlation coefficient
IOP: Intraocular pressure
IRB: Institutional Review Board
LoA: Limits of agreement
NCT: Non-contact tonometers
N-IOP: Intraocular pressure values obtained with the Nidek NT-530P
SD: Standard deviation
T-IOP: Intraocular pressure values obtained with the Topcon CT-1P
